# The Extent and Structure of Peri-urban Smallholder Dairy Farming in Five Cities in India

**DOI:** 10.3389/fvets.2020.00359

**Published:** 2020-07-03

**Authors:** Johanna F. Lindahl, Abhimanyu Chauhan, J. P. S. Gill, Razibuddin Ahmed Hazarika, Nadeem Mohamed Fairoze, Delia Grace, Abhishek Gaurav, Sudhir K. Satpathy, Manish Kakkar

**Affiliations:** ^1^Department of Biosciences, International Livestock Research Institute, Nairobi, Kenya; ^2^Department of Clinical Sciences, Swedish University of Agricultural Sciences, Uppsala, Sweden; ^3^Zoonosis Science Centre, Uppsala University, Uppsala, Sweden; ^4^Public Health Foundation India, Gurgaon, India; ^5^Department of Public Health Sciences, Faculty of Medicine, University of Liège, Liege, Belgium; ^6^Guru Angad Dev Veterinary and Animal Sciences University, Ludhiana, India; ^7^Department of Veterinary Public Health, Assam Agricultural University, Guwahati, India; ^8^Department of LPT, Veterinary College, Karnataka Veterinary Animal and Fisheries Sciences University, Bengaluru, India; ^9^Department of Veterinary Public Health and Epidemiology, College of Veterinary and Animal Science, Rajasthan University of Veterinary and Animal Sciences, Udaipur, India; ^10^School of Public Health, Kalinga Institute of Industrial Technology (KIIT) University, Bhubaneswar, India

**Keywords:** urban agriculture, food security, dairy production, South Asia, urban livestock keeping

## Abstract

Livestock keeping is common in many cities in India, driven by the demand for animal-source foods, particularly perishable milk. We selected five cities from different regions of the country and conducted a census in 34 randomly selected peri-urban villages to identify and describe all smallholder dairy farms. In total 1,690 smallholder dairy farms were identified, keeping on average 2.2 milking cows and 0.7 milking buffaloes. In Bhubaneswar, the proportion of cows milking was only 50%, but in other cities it was 63–73%. In two of the five cities, more than 90% of the farmers stated that dairy production was their main source of income, while <50% in the other cities reported this. In one of the cities, only 36% of the households kept milk for themselves. Market channels varied considerably; in one city about 90% of farms sold milk to traders, in another, 90% sold to the dairy cooperative, and in another around 90% sold directly to consumers. In conclusion, peri-urban dairy systems in India are important but also varying between different cities, with only one city, Bengaluru, having a well-developed cooperative system, and the northeastern poorer region being more dependent on traders. Further studies may be needed to elucidate the importance and to design appropriate developmental interventions.

## Introduction

India has a population of more than one billion people, and cities continue to expand, engulfing neighboring areas (1, 2). Urban inhabitants, as well as the growing middle-income classes, are increasingly demanding animal-source foods (3). While food may be brought from a far, animal-source foods are by nature perishable, and this creates a market for urban livestock keeping, especially in countries with tropical climates and poor infrastructure.

Urban and peri-urban livestock keeping is not new: it can be traced back to the origin of cities and perhaps even earlier (4). With time, however, the view of animals has changed; in the modern era many countries have worked actively to remove livestock from cities, with more or less success depending on the enforcement of regulations (4). In addition, urban and peri-urban livestock systems often supply informal markets, which may be banned or neglected in many low and middle-income countries, despite the fact that they are often the dominant market. The general view that livestock keeping is a rural practice has also led to an unequal distribution of resources, with most development resources going to improving rural production, extension services are provided to rural areas, livestock keeping is encouraged and supported, while in the urban and peri-urban production system, it is often neglected or even banned and subject to harassment. Worldwide concerns about food security, urbanization and local food production, have however contributed to bringing urban livestock keeping back on the agenda (5).

Livestock are important for the poor in many cities, and, in addition to securing food and nutrition for the urban population, and providing livelihoods for actors along the value chains, they also fill a niche by providing a productive use for food wastes and foods not deemed suitable for people (6, 7). Many different species are kept in urban areas, with species depending on cultural and religious preferences. When ruminants are kept in cities, it is mainly for milk production, and in India, where the population of cattle and buffaloes is the largest in the world (8), dairy production is particularly important in urban and peri-urban areas.

While the importance and constraints to urban and peri-urban livestock keeping in general have been reviewed extensively previously (9, 10), the objective of this paper is to demonstrate the extent of peri-urban dairy farming in five Indian cities to illustrate the importance of this.

## Methods

In order to estimate the extent of peri-urban smallholder dairy farming and establish a farmer census in a subset of villages, surveys were conducted in five Indian cities; Bengaluru, Bhubaneswar, Guwahati, Ludhiana, and Udaipur. The cities were selected to represent different regions of India, as well as different demographic sizes of cities (Table 1). In each city, the aim was to create a census of 34 villages, to assess the number of farms, but also to create a sampling frame for future surveys.

**Table 1:** Population (as of 2016) and peri-urban farms identified in five Indian cities.

				**Number of peri-urban small-scale dairy farmers**	
**City**	**State**	**Population**	**Literacy level (%)**	**Total identified**	Assumed total number^*^
Bengaluru	Karnataka	8,499,399	90	762	3,250
Bhubaneswar	Odisha	881,988	93	124	817
Guwahati	Assam	968,549	91	130	130
Ludhiana	Punjab	1,613,878	85	320	828
Udaipur	Rajasthan	475,150	90	354	791
Total		12,438,964	89	1,690	5,816

* *Calculated assuming that the selected 34 villages were similar to all peri-urban villages, and thus represented 34/total number of villages. In Guwahati, it was only assumed to be farming in the villages visited*.

Since peri-urban is not a term with an official definition, we define it as locations outside the official municipality city limits, as stated by the municipality corporation of the respective cities during census 2011, but within 5 km of these. A map was created by geotagging all villages within this fringe for each site. The total number of villages were tallied i.e., Udaipur-76, Bengaluru-145, Guwahati-127, Ludhiana-88, and Bhubaneswar-224. In each city, 34 villages were selected by systematic random sampling, by determining the proportion of villages to be selected, randomly selecting a start number, and after that, in a clock-wise fashion, visiting every nth village. If a village had less than three farms practicing smallholder dairying (defined as having <10 cows), the next village on the list was approached. In Guwahati, the administrative villages, “tolas,” were much larger than in the other cities, and comprised several smaller units, each with their own village head. Herein the entire peri-urban fringe region, a total of 22 “tolas” were identified that had dairy keeping, and thus only these were visited.

In the village, the village head was asked to provide a list of dairy farmers, which were keeping at least one cow or buffalo. Due to strong presence of Karnataka Milk Federation (KMF) in Bengaluru, the KMF secretary was approached for this, since KMF have milk collection centers in each village with lists of farmers. All dairy farmers in the villages were then visited, no matter the farm size. To ensure that a census was accomplished, this approach was complimented with a snowballing approach, where all farmers were also asked to identify other farmers they knew.

When a farm was identified, the owner, or another person in charge, was interviewed. Questions covered the number of people in the household, the livestock kept, and what was done with the milk produced. The farmers were also asked about the numbers of pigs, small ruminants, chicken and ducks kept in the farm. In Ludhiana, the data collection team reported only the number of cattle, and the other animals were handled as missing data. An estimate of the total number of farms in the peri-urban fringe was calculated assuming that the selected 34 villages were similar to all peri-urban villages, and thus represented 34/total number of villages. In Guwahati, it was only assumed to be farming in the villages visited. Descriptive data analyses were done in Excel and STATA 14.0 (StataCorp Ltd).

The study had ethical approval from the ethics committee of the Public Health Foundation of India [approval number TRC-IEC-219/14], as well as by Institutional Ethics Committees of Guru Angad Dev Veterinary and Animal Sciences University (GADVASU, Ludhiana), Assam Agriculture University (AAU, Guwahati), Karnataka Veterinary, Animal and Fisheries Sciences University (KVAFSU, Bengaluru), Rajasthan University of Veterinary and Animal Sciences (RAJUVAS, Udaipur) and School of Biotechnology, Kalinga Institute of Industrial Technology (KSBT, Bhubaneswar). Farmers were informed about the purpose of the study and gave consent before being interviewed.

## Results

In 34 villages in each of the four cities, and 22 “tolas” in one city, a total of 1,690 peri-urban dairy producers were identified and interviewed. Almost half of the farmers (45%) were identified in Bengaluru. In most farms (86%) the owner himself answered, while in the rest of the cases the interview was answered by a relative, most commonly the wife. In total, 86% of the respondents were male. Family composition is shown in Table 2.

**Table 2:** Household composition in peri-urban dairy farms in five Indian cities.

	**Adult males**	**Adult females**	**Male children**	**Female children**
Bengaluru	2.2 (0–12)	2.0 (0–8)	0.6 (0–7)	0.5 (0–6)
Bhubaneswar	2.9 (1–8)	2.8 (1–13)	0.8 (0–6)	0.6 (0–6)
Guwahati	2.4 (1–5)	2.3 (0–6)	0.6 (0–3)	0.6 (0–5)
Ludhiana	2.4 (1–7)	2.3 (0–9)	0.8 (0–5)	0.5 (0–5)
Udaipur	1.2 (0–4)	1.2 (0–5)	0.1 (0–3)	0.1 (0–4)

Most of the respondents stated that they owned the land of the farm. In Bengaluru and Udaipur, all farmers said they owned the land, similar to 0.8% in Bhubaneswar, and 0.9% in Ludhiana, but there were significantly more (*p* < 0.01), 12%, in Guwahati reporting that they leased the land.

Most farms (76%) both sold milk and used for household consumption. Of the rest, half used it only for selling, and the other half only for household consumption. Out of the 209 households that did not sell milk, 205 were in Udaipur. Families that did not keep milk for household consumption had the same average number of children as those who did, with up to eight children in a household. For 48% of the households, the dairy farm was the major source of income, but there were significant differences (*p* < 0.001) between the sites, with much higher proportions reporting this in Bhubaneswar and Guwahati (Table 3). There were also differences concerning which actor bought the milk. In total, only 13 farmers sold milk to more than one type of actor, most of these were in Bengaluru and Ludhiana. The lowest proportion of milking cows was in Bhubaneswar where 50% of cows kept were milking (Table 4), while the other sites had significantly higher percentages (between 63 and 73% milking cows, *p* < 0.01). In all the three sites where peri-urban farmers kept buffaloes, the proportion of milking buffaloes exceeded 68%.

**Table 3:** The sale of milk to different customers among peri-urban dairy farmers in five Indian cities.

**City**	**Dairy is the major source of income (%)**	**Consume themselves (%)**	**Sell milk (%)**	**Sell to milk cooperative (%)**	**Sell to consumers (%)**	**Sell to traders (%)**
Bengaluru	35	99	99	92	7.0	0.9
Bhubaneswar	95	36	100	11	89	0.8
Guwahati	94	100	100	0	10	90.0
Ludhiana	46	87	100	13	53	36.8
Udaipur	44	80	41	34	37	29.1
Total	48	88	88	55	27	19.1

**Table 4:** Dairy animals in peri-urban dairy farms in five Indian cities.

**City**	**Average cows (range)**		**Average milking cows (range)**		**Average buffaloes (range)**		**Average milking buffaloes (range)**	
Bengaluru	3.7	(0–30)	2.1	(0–15)	0.2	(0–40)	0.1	(0–25)
Bhubaneswar	5.4	(3–12)	2.7	(1–10)	0.1	(0–5)	0.0	(0–0)
Guwahati	7.2	(1–22)	4.4	(1–10)	0.0	(0–0)	0.0	(0–0)
Ludhiana	1.3	(0–20)	0.9	(0–12)	3.4	(1–12)	2.4	(1–8)
Udaipur	3.4	(1–50)	2.4	(0–32)	2.1	(1–12)	1.4	(1–10)
Total	3.6	(0–50)	2.2	(0–32)	1.1	(0–40)	0.7	(0–25)

Less than 1% of the households also kept pigs. In Bengaluru, 23.9% of the dairy farmers also kept poultry, in Guwahati 5.4% and in Udaipur 4.2%, while only 0.8% of farms in Bhubaneswar had poultry. Ducks were only present in Bengaluru and Guwahati, where 2.2 and 5.4%, respectively, of households reported having them. There was a large variation in households having small ruminants. More households kept small ruminants in Bengaluru (23.4%, average 8.1 animals), Guwahati (20.8%, average 3.9 animals), and Udaipur (20.6%, average 6.4 animals) compared to Bhubaneswar where only 11.3% of households kept small ruminants, but in those farms, the average number of animals kept was 20.6.

## Discussion

This study showed the prominence of dairy farming in the peri-urban areas in India, with 1,690 smallholder dairy households identified throughout the five cities, and potentially almost 6,000 peri-urban farmers present around the five cities, assuming that all villages had the same proportion of cattle-keeping. This corresponds to around one in 534 households being led by a dairy farmer. However, it is considerably lower than reported from some east African farmers: an estimated one in 80 households in peri-urban Nairobi keep dairy cattle and one in 90 households in Addis Ababa (11).

More than 90% in Bhubaneswar and Guwahati depended on dairy production for their income. This shows how important peri-urban farming can be for the food production as well as for family livelihoods in India. In Ludhiana and Udaipur, dairy was the major source of income for just under half the farmers and in Bengaluru for just over one third.

There were many differences among the cities. In Bhubaneswar and Guwahati, there were only reports of up to 10 milking cows among the visited households, which for Guwahati is lower than previously reported numbers (12). There were also no households with buffaloes in Guwahati, and very few in Bhubaneswar. Compared to the rest of India, the northeastern state of Assam, has had slower development of the dairy sector than the rest of the country, and the availability of milk per person was much lower in 2013–14, 69 g/day, than the Indian average, 307 g/day (13). Unsurprisingly, fewer dairy farms were found in Guwahati, and the peri-urban dairying was clustered. The average number of cows per farm was higher, with on average more than 4 milking cows, and all farms sold milk and consumed it in the household. In Guwahati the sale of milk was dependent on traders, through which 90% sold their milk. The importance of milk traders in Guwahati, along with low productivity in dairy farms and low knowledge among all dairy value chain actors, has been observed in earlier studies as well (12, 14). The difference between Guwahati and the other cities has also been demonstrated when it comes to disease prevalence (15).

In Udaipur, Rajasthan, farmers were almost equally likely to sell to traders, consumers, or cooperatives. Bengaluru, in Karnataka, had strong dairy cooperatives, to which more than 90% of farmers sold their milk. This may be the result of the Karnataka Dairy Development Project which promoted cooperatives, and villages that participated in this produced more milk (16). Dairy cooperatives have been described as important components for developing dairying in tropical countries (17), but it was only in Bengaluru that we found this system to have a dominant role. However, <40% stated that dairy was the main source of income in this city, and more than 20% also kept poultry, in difference to the other cities, where poultry keeping was less common.

Ludhiana, in Punjab, had most buffaloes, and more than half the farmers sold directly to consumers. Consumers were also the most common buyer in Bhubaneswar, Odisha. Here, the fact that less than half of the cows kept were milking implies that there are large productivity losses. Key performance indicators for tropical dairy farmers state that the target proportion of cattle milking should be >73% (17), which most of the farmers in this study did not reach.

We found that <40% of farms in Bhubaneswar would keep milk for household consumption. In spite of the many positive nutritional aspects of milk, and the increased potential of dairy keeping households to provide milk to the children (18), around 200 households reported only selling the milk and not using it for household consumption, which is a concern, especially when there are children in the households. Milk is an important source of high biological value protein, as well as micro- and macronutrients, and especially important for children. In spite of being relatively cheap, many children in low and middle-income countries consume less than recommended amounts (19). Especially in a country with a large vegetarian population like India (20), milk is of great importance and dairy products may be the sole source of animal proteins for many children. The results of this study highlight the needs to reach also dairy farmers with more messages on the benefits of keeping milk for children in the family. In Karnataka, an earlier study found that the lowest milk consumption in children were in households not producing milk at all (21), and it could also be beneficial to increase the knowledge levels of parents overall of the benefits of milk to children.

Approximately 70 million Indian households are believed to be engaged in dairy production (22), and while the assumptions for calculating a potential number of dairy farms in the peri-urban area of these cities, our estimates are likely an underestimation of the total number of farmers, and a total census may be warranted to get the total number. This study however indicate that the peri-urban belts around the large cities may be very important for the food supply and comprise a significant number of smallholder dairy farmers.

## Data Availability Statement

The datasets generated for this study are available on request to the corresponding author.

## Ethics Statement

The studies involving human participants were reviewed and approved by the ethics committee of the Public Health Foundation of India, approval number TRC-IEC-219/14, May 27, 2014; amended Oct 12, 2015. Written informed consent to participate in this study was provided by the farmers.

## Author Contributions

JL drafted the manuscript and all other authors reviewed and contributed. JL, DG, and MK designed the study with input from all authors. AC coordinated data collection, with support from NF, RH, and JG. All authors contributed to the article and approved the submitted version.

## Conflict of Interest

The authors declare that the research was conducted in the absence of any commercial or financial relationships that could be construed as a potential conflict of interest. The handling Editor declared a shared affiliation, though no other collaboration, with one of the authors JL.
